# A negative role for the interleukin-2-inducible T-cell kinase (ITK) in human Foxp3^+^ T_REG_ differentiation

**DOI:** 10.1371/journal.pone.0215963

**Published:** 2019-04-25

**Authors:** Polina Mamontov, Ryan A. Eberwine, Jackie Perrigoue, Anuk Das, Joshua R. Friedman, J. Rodrigo Mora

**Affiliations:** Janssen Research & Development, Spring House, Pennsylvania, United States America; McGill University Health Centre, CANADA

## Abstract

The Tec kinases ITK (interleukin-2-inducible T-cell kinase) and RLK (resting lymphocyte kinase) are critical components of the proximal TCR/CD3 signal transduction machinery, and data in mice suggest that ITK negatively modulates regulatory T cell (T_REG_) differentiation. However, whether Tec kinases modulate T_REG_ development and/or function in human T cells remains unknown. Using a novel self-delivery siRNA platform (sdRNA), we found that ITK knockdown in human primary naïve peripheral blood CD4 T cells increased Foxp3^+^ expression under both T_REG_ and T helper priming conditions. T_REG_ differentiated under ITK knockdown conditions exhibited enhanced expression of the co-inhibitory receptor PD-1 and were suppressive in a T cell proliferation assay. ITK knockdown decreased IL-17A production in T cells primed under Th17 conditions and promoted Th1 differentiation. Lastly, a dual ITK/RLK Tec kinase inhibitor did not induce Foxp3 in CD4 T cells, but conversely abrogated Foxp3 expression induced by ITK knockdown. Our data suggest that targeting ITK in human T cells may be an effective approach to boost T_REG_ in the context of autoimmune diseases, but concomitant inhibition of other Tec family kinases may negate this effect.

## Introduction

Interleukin-2-inducible T-cell kinase (ITK) is a member of the Tec kinase family of non-receptor tyrosine kinases and mediates T cell signaling downstream of TCR activation [1]. Signaling through ITK modulates T cell activation, T helper cell differentiation, and thymic selection of developing thymocytes. ITK has been implicated as a critical node in T cell and NK cell mediated inflammation, leading to interest in developing therapeutics to modulate ITK function in autoimmune and inflammatory diseases [2, 3]. ITK is thought to drive Th2-mediated disease such as allergic asthma, and ITK^-/-^ mice exhibit significantly improved disease course and reduced bronchoconstriction after antigen re-challenge in ovalbumin sensitized mice [2, 4]. ITK has also been shown to regulate the balance between inflammatory CD4^+^ Th17 cells and CD4^+^ Foxp3^+^ regulatory T cells (T_REG_) in mice [5]. In addition, ITK is an important switch for Th1 and Th2 mediated immunity, and murine ITK deficiency results in reduced differentiation and effector cytokine production from Th1, Th2, and Th17 polarized CD4^+^ T cells, while bolstering T_REG_ development [5–8]; in contrast, some data suggest that ITK deficiency increases Th1 differentiation under some conditions [9]. However, since ITK is also involved in thymocyte development, studies in ITK knock-out mice may not distinguish potential developmental defects in the immune system from the effects of ITK inhibition on the mature immune system [10]. Although ITK also serves a non-kinase ‘scaffolding’ function for the docking of signaling intermediates [11], studies in kinase-dead ITK mutant mice have shown that kinase activity is required for driving Th1, Th2, and Th17 differentiation [6, 7], suggesting that a specific kinase-inhibitor may modulate ITK effects on T cell differentiation.

Resting lymphocyte kinase (RLK) is another member of the Tec family of non-receptor tyrosine kinases closely related to ITK. While less is known about RLK in T cell signaling and differentiation, both ITK and RLK are activated by Src kinases downstream of the TCR signaling complex [12]. On the other hand, RLK is constitutively bound to the T cell plasma membrane via an N-terminal palmitoylation site, whereas ITK has a pleckstrin homology domain which requires PI3K-mediated PIP3 generation for recruitment to the plasma membrane after TCR activation [12–15]. In addition, ITK^-/-^ mice exhibit impaired CD4^+^ and CD8^+^ T cell development, whereas RLK deficiency alone does not affect T cell development. However, mice deficient in both ITK and RLK have a marked defect in T cell activation in response to anti-CD3, which can be bypassed by activating a downstream PKC with phorbol 12-myristate 13-acetate (PMA) [1].

While ITK is required for IL-17A production in human T cell lines [14] and regulates Th17 and T_REG_ differentiation in mice [5], its role in human T_REG_ differentiation is not defined. Here we investigated the roles of ITK in human Foxp3^+^ T_REG_ differentiation and function using self-delivered siRNA (sdRNA) optimized to decrease ITK expression in resting primary human T cells. We found that ITK is a negative regulator of human T_REG_ differentiation under *in vitro* T_REG_, Th17, and Th1 polarizing conditions, and that ITK reciprocally regulates T_REG_ and Th17 differentiation from naïve human CD4^+^ T cells. Moreover, we show that ITK knockdown upregulates the expression of the co-inhibitory molecule PD-1 on *in vitro*-differentiated human Foxp3^+^ CD4^+^ T cells. Pharmacologic inhibition of both ITK and RLK blocked the T_REG_ bolstering effect of ITK knockdown, suggesting that RLK may be required for T_REG_ differentiation in the absence of ITK. Our data show for the first time that ITK regulates CD4^+^ T_REG_ and Th17 differentiation in primary human T cells, and highlights the potential for pharmacological targeting of specific Tec kinases in inflammatory and autoimmune disease.

## Materials and methods

### Cell culture

Naïve CD4^+^ CD45RA^+^ CD25- peripheral blood T cells were purchased from AllCells (Alameda, CA). For Th0 non-polarized condition, T cells were cultured in RPMI 1640 media with penicillin/streptomycin and 10% HI-FCS, and 1 μg/ml of anti-CD28 on wells coated with 1 μg/ml anti-CD3 (OKT3, eBioscience). For T cell polarizing conditions T cells were supplemented as follows. T_REG_: 300 U/ml IL-2, 5 ng/ml rhTGFβ. Th17: 10 μg/ml anti-INFγ, 10 μg/ml anti-IL-4, 20 U/ml rhIL-2, 10 ng/ml IL-23, 0.5 ng/ml TGFβ, 10 ng/ml IL-1β. Th1: 10 μg/ml anti-IL4, 20 U/ml IL-2, 10 ng/ml IL-12. Cytokines were from R&D systems (Minneapolis, MN) and antibodies from eBioscience (San Diego, CA).

### Knock down of ITK gene expression

Self-delivery siRNA (sdRNA) constructs were purchased from Advirna (Cambridge, MA). CD4 T cells were cultured in 1% serum in RPMI 1640 under non-activating conditions with the addition of sdRNA as per the manufacturer’s specifications. After 24h the concentration of serum was increased to 10%, and 24h later T cells were transferred to media conditioned for T_REG_, Th17, Th1, or Th0 differentiation. Quantitative RT-PCR (qPCR) analysis was performed at a range of time points post-sdRNA addition to assess the extent of ITK knockdown. T cells were analyzed after 4 days of culture under polarizing conditions by flow cytometry for Foxp3 (T_REG_), CCR6 (Th17), and IFNγ (Th1) expression. Several ITK-targeting sdRNA constructs were assessed in comparison to a non-targeting control (NTC) for ITK knock-down, and one construct (ITK49, henceforth sd-ITK) was selected based on optimal ITK knockdown efficiency.

### *In vitro* suppression assay

CD4 T cells were cultured under T_REG_ conditions (T_REG_-polarized) with either NTC or sd-ITK. Four days later T_REG_-polarized cells were collected and labeled with CellTrace Violet (CVT, Life Technologies). Peripheral blood responder CD4 T cells (AllCells; Alameda, CA) were labeled with CFSE (T-responder). T_REG_-polarized and T-responder were cultured with anti-CD3/CD28 activation beads (1:10 bead:T cell ratio; Miltenyi Biotec). Cells were co-cultured at different T_REG_-polarized/T-responder cell ratios (from 1:8 to 1:1) for 72h. After that, the cells were fixed and analyzed by flow cytometry to determine T cell proliferation based on CFSE dilution. Proliferation profile and division index analyses were performed using FlowJo.

### qRT-PCR analysis

Total RNA was isolated from cultured T cells with the RNeasy 96 kit (Qiagen; Germantown, MD) and reverse transcribed into cDNA with qScript cDNA mastermix (Quanta Biosciences; Gaithersburg, MD). qPCR was performed with Taqman primer-probe sets and Taqman universal mastermix (Applied Biosystems). Data were collected on the Viaa7 Real-Time PCR system (Applied Biosystems^™^) and analyzed by the comparative Ct method (ΔΔCt) with normalization to the mean Ct of endogenous control genes HPRT1, TBP, and IPO8.

### Flow cytometry

Cells were stained with the human regulatory T cell staining kit #1 (eBioscience) using anti-CD4, anti-CD25, anti-Foxp3, and anti-CCR6 antibodies. Prior to intracellular IFNγ staining, T cells were stimulated with PMA/Ionomycin for 5h and in the presence of Monensin for the last 4h of stimulation. Cells were analyzed by flow cytometry using FlowJo software.

### Luminex

Cytokines in culture media supernatants were measured with the Luminex platform using the Milliplex Human Cytokines 38 plex Immunology Assay (Millipore-Sigma; Billerica, Massachusetts).

### ITK/RLK kinase inhibitor compound

The dual ITK/RLK small molecule kinase inhibitor was synthesized in-house (JNJ64461449) based on published data [2, 3]

### Statistics

Results are expressed as scatter dot plots, including median ± interquartile range (IQR). For most analyses the raw data were log10-transformed to approximate a normal distribution. Student’s t test was used when comparing two groups. One-way ANOVA with Dunnet’s post-hoc test or multiple t-tests with adjustment for multiple testing (two-stage step-up method of Benjamin, Krieger, and Yekutieli) was used when comparing more than two groups. Paired analyses were done whenever appropriate. Graphs and analyses were generated with Prism-GraphPad software v8.0.2.

## Results

### ITK knockdown in human naïve CD4 T cells promotes Foxp3+ upregulation upon activation

**Fig 1:**
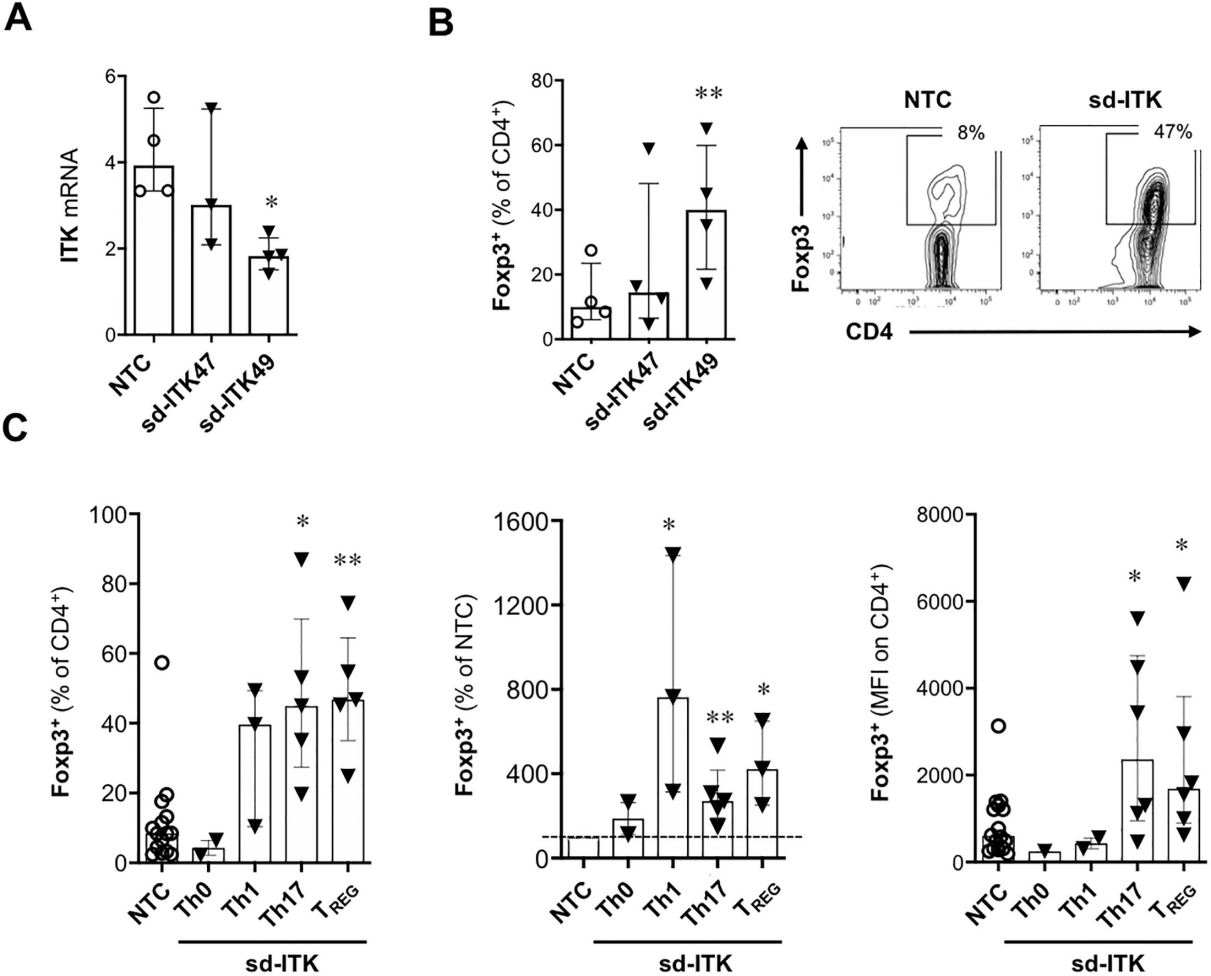
ITK knock-down promotes increased Foxp3 expression in human CD4 T cells activated under different T cell polarizing conditions. (A) ITK mRNA expression in sdRNA-treated cells was normalized by the ΔΔCt method to the mean of endogenous control genes (TBP, IPO8, and HPRT1). CD4 T cells were activated in the presence of a non-targeting control (NTC) sdRNA or two ITK-targeting sdRNA constructs. sd-ITK49 (henceforth sd-ITK) showed higher knockdown efficiency compared to sd-ITK47 and was therefore exclusively used in most subsequent experiments. N = 5 donors. (B and C) Human naïve CD4^+^ T cells were treated with NTC or ITK targeting sdRNAs and then activated under Th17, Th1, or T_REG_ polarizing conditions. Four days later, CD4 T cells were analyzed for Foxp3 expression by flow cytometry. N = 3–5 donors per condition. (B) Foxp3 expression in CD4 T cells activated under T_REG_-polarizing conditions in the presence of NTC or two ITK-targeting sdRNA constructs. Right panels show representative flow cytometry plots of T cells treated with NTC or sd-ITK. (C) Foxp3 expression in CD4 T cells activated under different polarizing conditions in the presence of NTC or sd-ITK. Median ± IQR, *p<0.05, **p<0.01 (paired one-way ANOVA with Dunnett’s post-hoc test vs. NTC).

To assess the effect of ITK on human T_REG_ differentiation, we used self-delivered siRNA (sdRNA; Advirna, Cambridge, MA) which circumvents the low efficiency of conventional gene knockdown methods in resting primary human T cells. ITK knockdown was optimized using several sdRNA constructs; sd-ITK47 did not consistently downregulate ITK mRNA, whereas sd-ITK49 (henceforth sd-ITK) led to a 50%-80% reduction in ITK mRNA expression relative to a non-targeting sdRNA control (NTC) in human peripheral blood naïve CD4^+^ T cells (Fig 1A). An inverse relationship between ITK knockdown and Foxp3 expression was observed in T_REG_-polarized CD4 T cells (Fig 1A and 1B). ITK knockdown under T_REG_, Th17, Th1, or non-polarizing conditions (Th0) resulted in an increase in the frequency of Foxp3^+^ T cells, especially in the T_REG_ and Th17 polarizing conditions (Fig 1C**)**. These results suggest that ITK negatively regulates Foxp3 expression in human CD4 T cells.

### ITK knockdown modulates Th17 and Th1 differentiation in human T cells

**Fig 2:**
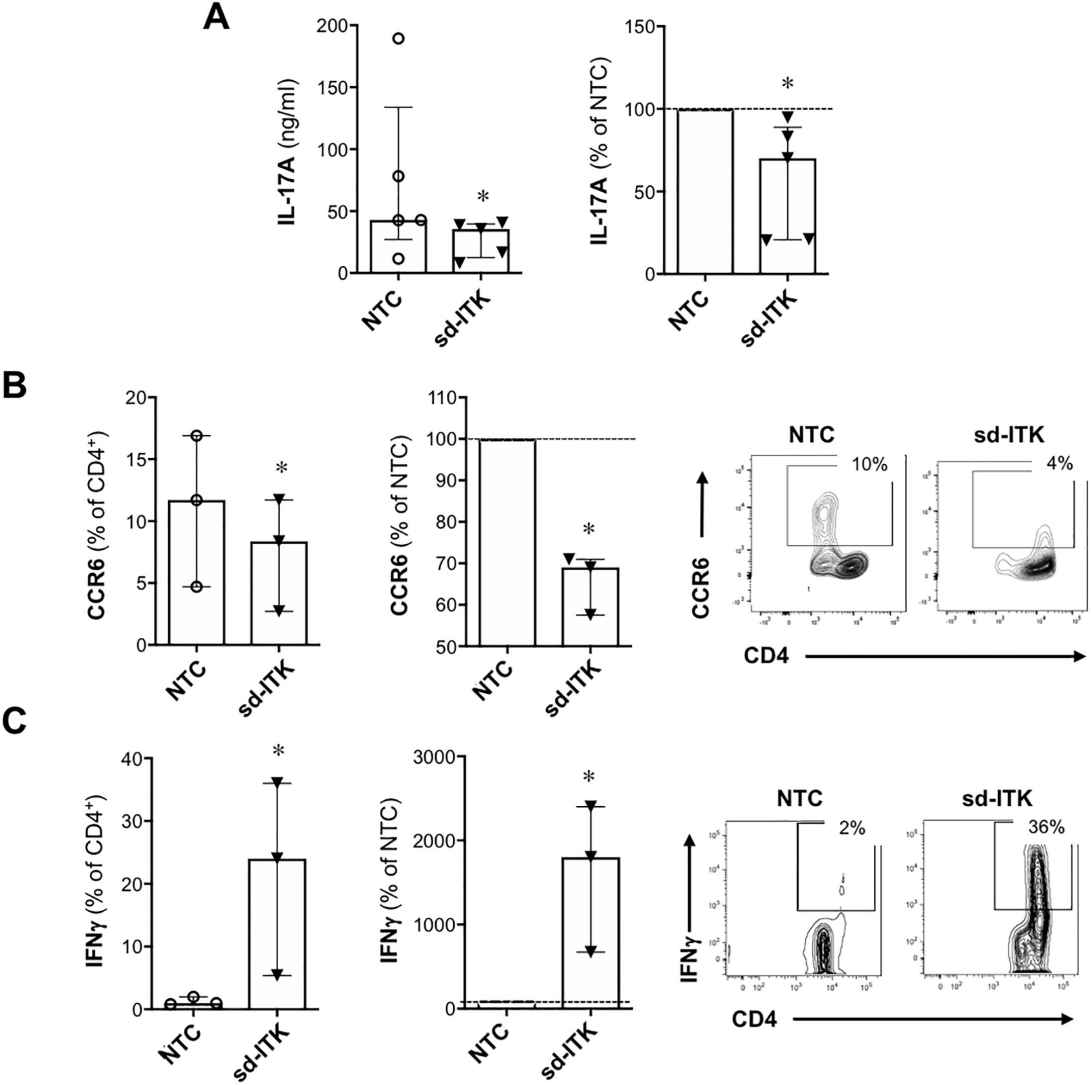
ITK knock-down impairs Th17 and promotes Th1 differentiation. Human naïve CD4 T cells were treated with NTC or sd-ITK and then activated under Th17 or Th1 polarizing conditions. Four days later culture supernatants were assessed for IL-17A by Luminex (A; N = 5), and CD4^+^ T cells were analyzed for CCR6 (B; N = 3) or INFɣ (C; N = 3) by flow cytometry. Right panels show representative flow cytometry plots of T cells treated with NTC or sd-ITK. Median ± IQR, *p<0.05 (paired two-tailed Student’s t-test).

Th17 cells produce the cytokine IL17A and express the chemokine receptor CCR6. We examined IL17A secretion and surface expression of CCR6 in human Th17-polarized cells upon ITK knockdown and observed down-regulation of both markers relative to T cells treated with NTC (Fig 2A and 2B). Thus, analogous to mouse T cells [5], ITK reciprocally regulates the differentiation of T_REG_ and Th17 cells in human primary T cells.

While ITK is critical for driving Th2 mediated immune responses and effector cytokine production [8, 16, 17] some studies indicate that ITK is also required for Th1 effector cytokine production in mouse T cells [6, 8, 17]. However, we observed that ITK knockdown led to increased IFNγ expression (Fig 2C). This is in contrast to what has been observed in mice expressing a mutant kinase-dead form of ITK, where ITK kinase activity was required for Th1, Th2, and Th17 effector cytokine production [6]. Thus, the effect of ITK knockdown on Th differentiation might not necessarily be mirrored by modulating its kinase activity.

### ITK knockdown increases the proportion of human Foxp3^+^ CD4 T cells expressing the co-inhibitory receptor PD-1 (CD279) and promotes the differentiation of functional T_REG_.

**Fig 3:**
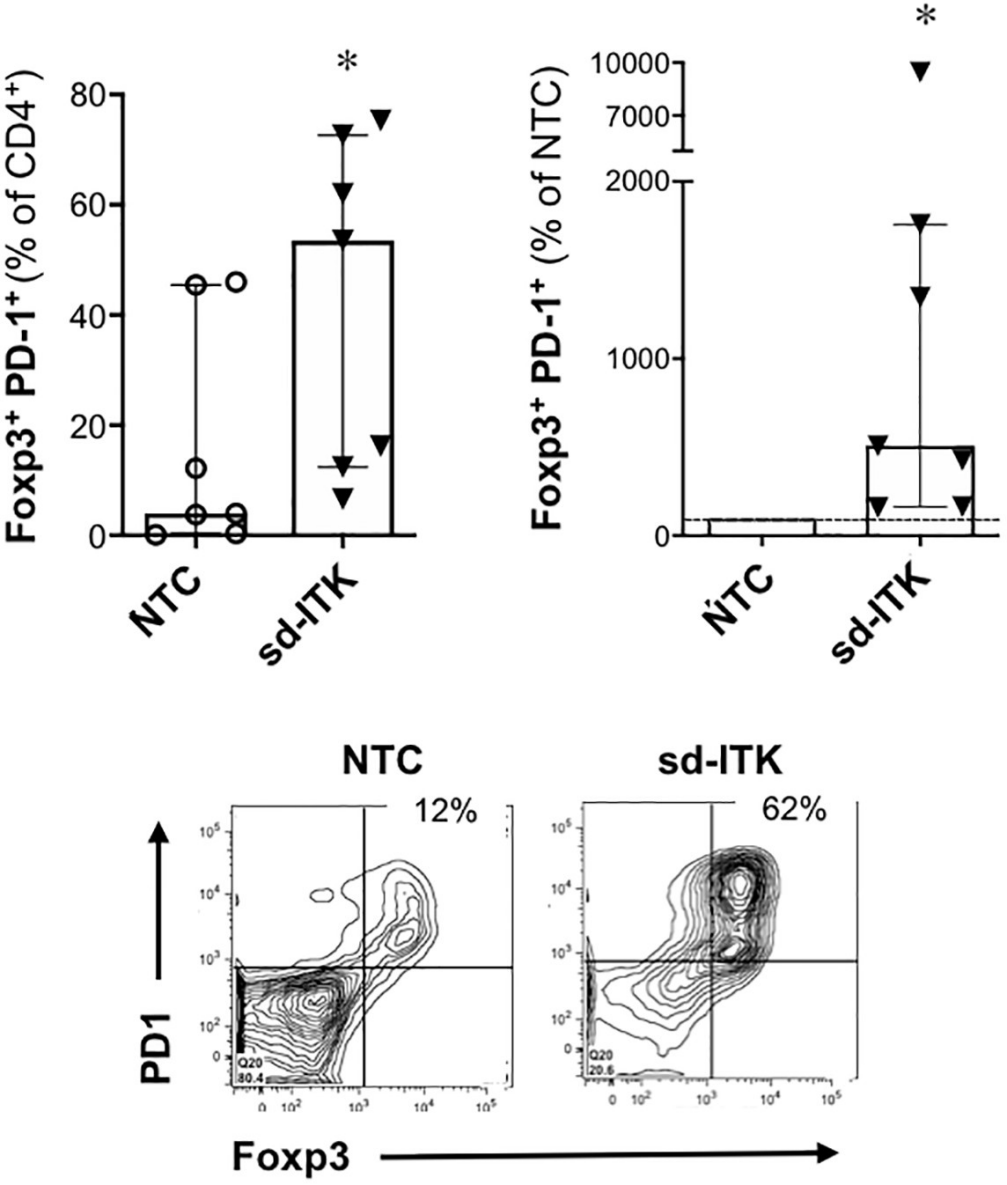
ITK knock-down increased differentiation of Foxp3^+^PD-1^+^ CD4 T cells. Human naïve CD4 T cells were treated with NTC or sd-ITK and then activated under T_REG_ polarizing conditions. Four days later CD4 T cells were analyzed for Foxp3 and PD-1 expression by flow cytometry. N = 7 donors. Lower panels show representative flow cytometry plots of T cells treated with NTC or sd-ITK. Median ± IQR, *p<0.05 (paired two-tailed Student’s t-test).

**Fig 4:**
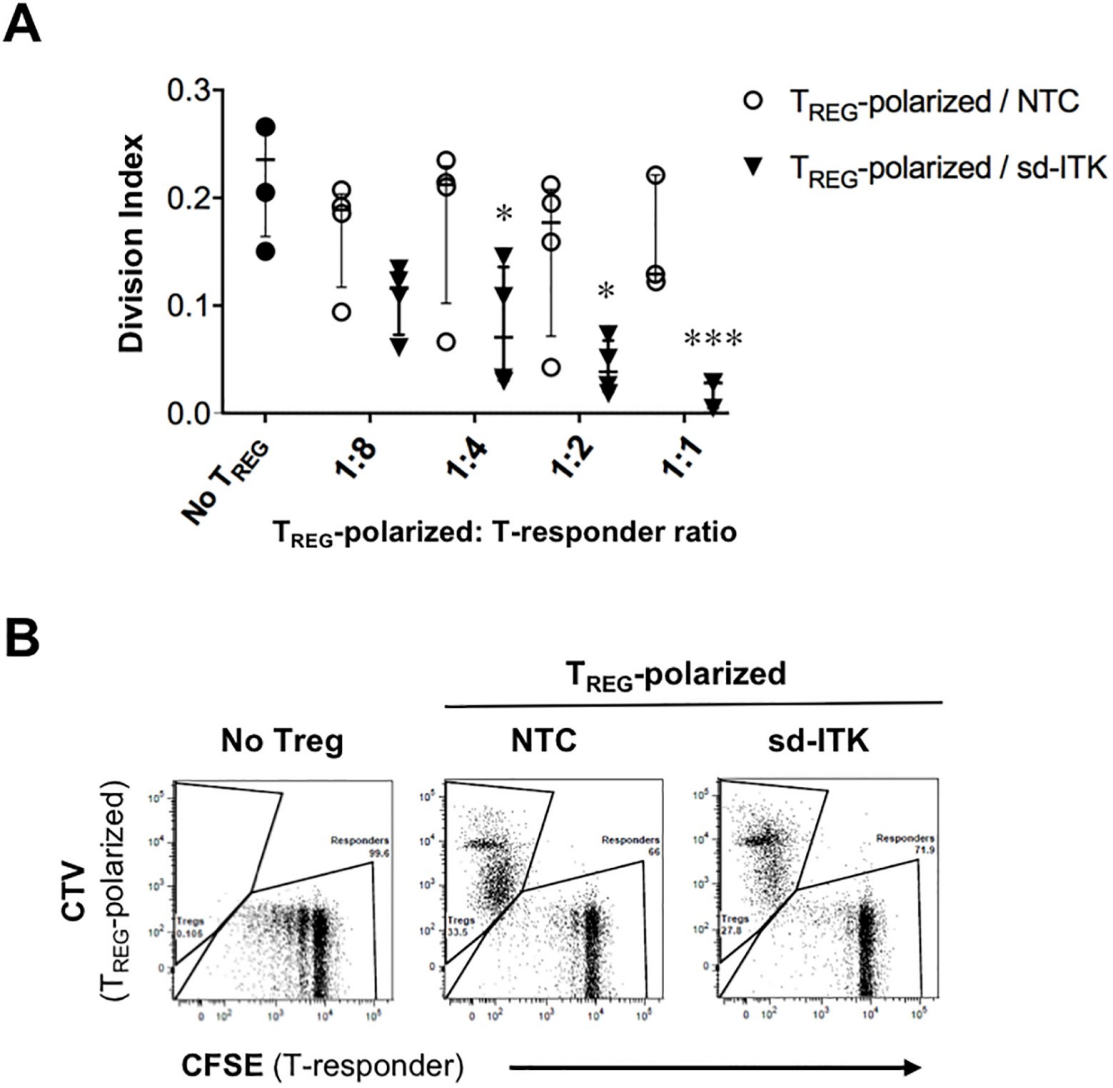
ITK knock-down induces bona-fide suppressive T_REG_. Human naïve T cells treated with NTC or sd-ITK and differentiated under T_REG_ polarizing conditions (T_REG_-polarized) were labeled with CVT, and CD4^+^CD25^Neg^ responder T cells were labeled with CFSE (T-responder). T_REG_ and T-responder were cultured in the presence of anti-CD3/CD28 activation beads. Flow cytometry analysis was performed after 4 days. (A) Division index for each T_REG_-polarized/T-responder ratio was calculated as the average number of cell divisions of the original CFSE-labeled population versus the undivided CFSE^high^ cells using the FlowJo proliferation platform. (B) Representative flow cytometry plots using a 1:1 T_REG_-polarized/T-responder ratio. N = 4 donors. Median ± IQR, *p<0.05, ***p<0.001 (multiple t-tests; see Methods).

While Foxp3 expression is required for T_REG_ differentiation, in humans it may not be sufficient to define functional T_REG_. Nonetheless, ITK knockdown led to a significantly higher increase in the proportion of Foxp3^+^ cells expressing the co-inhibitory molecule PD-1, suggesting that ITK knockdown promotes differentiation of bona fide T_REG_ (Fig 3). To directly assess this point, T_REG_-polarized cells in the presence of NTC or sd-ITK were co-cultured with CFSE-labeled CD4^+^ responder cells (T-responder) plus anti-CD3/anti-CD28 activation beads, and the effect on T-responder proliferation was measured by CFSE dilution. T_REG_-polarized sd-ITK cells showed a stronger and dose dependent effect on T cell proliferation compared to similar numbers of T_REG_-polarized NTC cells (Fig 4), which is consistent with the higher proportion of Foxp3^+^PD-1^+^ CD4 cells in T_REG_-polarized sd-ITK cells (Fig 3). While the data do not allow us to determine whether ITK knockdown enhances T_REG_ suppression on a per-cell basis, we conclude that the increased proportion of Foxp3^+^PD-1^+^ cells in T_REG_-polarized sd-ITK cells is correlated with overall increased suppressor activity, suggesting that ITK knockdown leads to the generation of bona fide functional human T_REG_.

### A pharmacological ITK/RLK kinase inhibitor does not upregulate Foxp3 and abrogates the effect of ITK knockdown on Foxp3 induction

**Fig 5:**
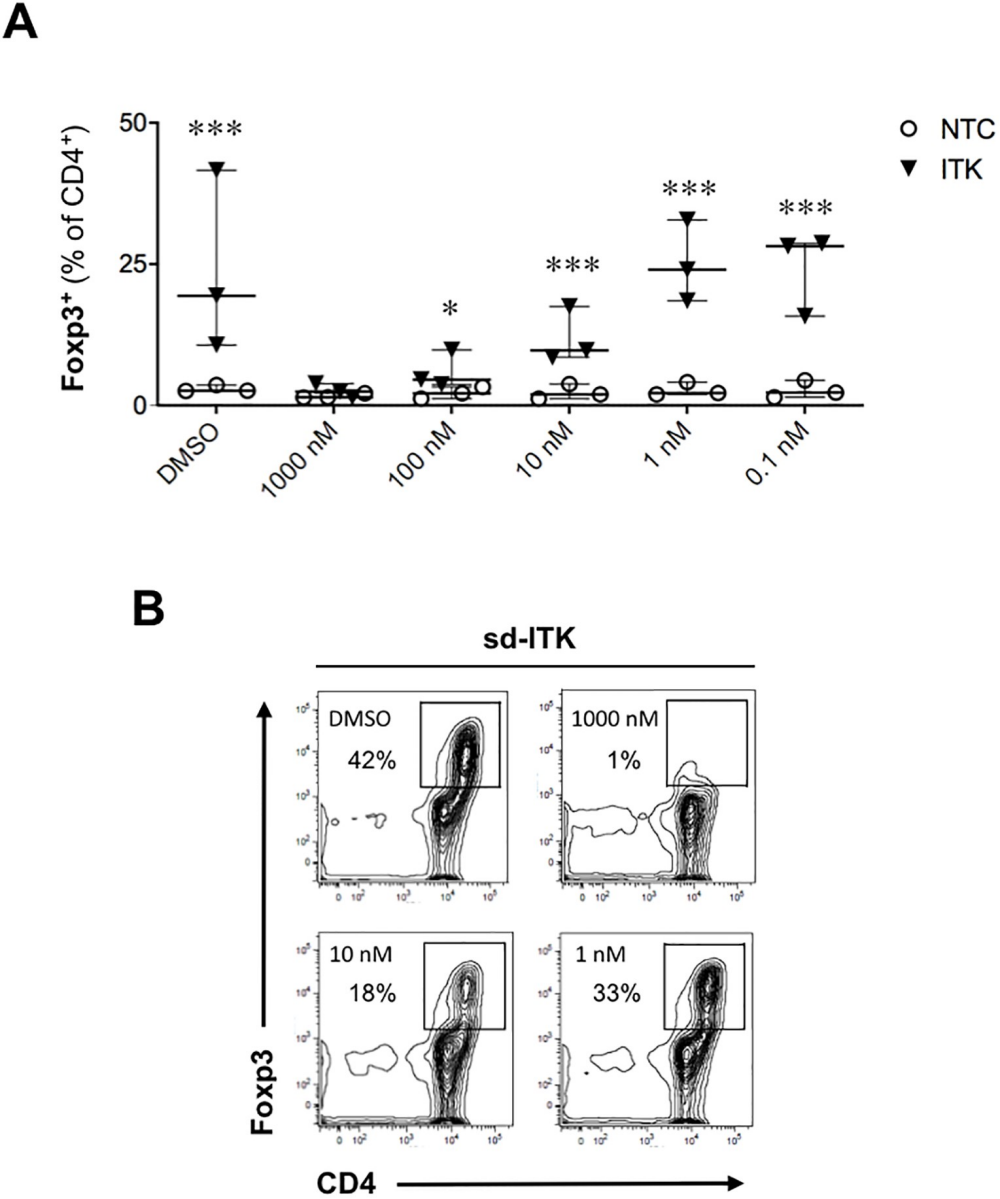
A pharmacological ITK/RLK kinase inhibitor does not upregulate Foxp3 and abrogates the effect of ITK knockdown on Foxp3 induction. Human naïve CD4 T cells were treated with NTC or sd-ITK followed by supplementation of the ITK/RLK kinase inhibitor PRN694 or DMSO as control. Cells were then activated under T_REG_ polarizing conditions, and four days later CD4^+^ T cells were analyzed for Foxp3 expression by flow cytometry. (A) T_REG_-polarized cells with NTC or sd-ITK in the presence of different concentrations of PRN694 or DMSO. N = 3 donors. (B) Representative flow cytometry plots showing the effect of PRN694 on Foxp3 expression induced by ITK knockdown. Median ± IQR, *p<0.05, ***p<0.001 (multiple t-tests; see Methods).

Studies in ITK deficient mice have fostered significant interest in targeting this kinase in autoimmune and inflammatory diseases [5, 6, 8, 18]. The covalent inhibitor PRN694 targets mainly ITK and RLK, and it blocks T cell activation, proliferation, and T helper cell differentiation [2, 3]. Furthermore, this compound ameliorated colitis progression in the T cell transfer colitis model by blunting both Th1 and Th17 responses [2]. However, in contrast to ITK-deficient mice, PRN694-treated mice exhibit reduced T_REG_ numbers, suggesting that RLK may play a distinct role in T_REG_ differentiation [2, 5]. In agreement with the findings in mice, PRN694 did not induce Foxp3 in human T cells, and it abrogated the increase in Foxp3 observed upon ITK knockdown in a dose-dependent manner (Fig 5), indicating that dual ITK/RLK kinase targeting is not permissive for T_REG_ differentiation.

Since RLK expression is suppressed after T cell activation [15], this Tec kinase is likely to be involved in early T cell activation events which are required to drive both T helper and T_REG_ differentiation. We attempted to isolate the effect of RLK on T_REG_ differentiation using sdRNA knockdown, but none of the eleven sdRNA constructs we tested alone or in combination reduced TXK gene expression. Thus, while it is possible that RLK and ITK play distinct roles in T_REG_ and T helper differentiation, at this point we cannot determine whether these Tec kinases play redundant or reciprocal roles in this regard.

## Discussion

The Tec family of tyrosine kinases, including ITK and RLK, are critical for TCR-mediated T cell activation and T helper cell differentiation, and they are likely to play distinct roles in these processes. We show here for the first time that ITK is a negative regulator of human Foxp3^+^ T_REG_ and Th1 development, with a reciprocal effect on Th17 differentiation. Our findings extend recent observations in ITK deficient mice, which display enhanced T_REG_ development *in vivo* and *in vitro* and diminished T effector development and function [5, 6, 8].

In addition, we observed that ITK knockdown led to an increase in IFNγ production by Th1 polarized cells. ITK has a well-established role in Th2 mediated inflammation, but reports are conflicting on its role in Th1 effector function [2, 6, 8, 9, 19, 20]. While some data indicate that ITK positively regulates Th1 development and effector function in mouse T cells [6, 8, 9, 20], our data suggest that ITK is dispensable for Th1 effector function in primary human T cells, and that targeting ITK alone is therefore unlikely to be broadly immunosuppressive.

While increased IFNγ production has been observed in T cells from ITK^-/-^ mice or upon ex vivo ITK knockdown [9], T cells from kinase-dead ITK mutant mice paradoxically showed a marked decrease in production/secretion of IFNγ and other cytokines upon TCR stimulation [8]. We hypothesize that a kinase-independent ‘scaffolding’ effect of ITK is involved on Th1/Th2 suppression; this scaffolding effect would be abrogated upon ITK knockdown, but not when using a specific pharmacological ITK kinase inhibitor, which would instead be expected to emulate the effects observed with the ITK kinase-dead mutant. Thus, while ITK knockdown led to increased IFNγ production, we predict that a specific ITK kinase inhibitor might not lead to a similar effect. Whether an analogous paradigm could apply to Foxp3 upregulation remains to be determined. The current lack of a specific ITK inhibitor precludes testing these potential outcomes.

Pharmacologic inhibition of both ITK and RLK in human naïve T cells blocked T_REG_ differentiation, suggesting that, in contrast to ITK, RLK might be required for T_REG_ differentiation. Although ITK and RLK are both activated upon TCR ligation, only ITK is dependent on the PI3K-AKT pathway [12], which is involved in the repression of Foxp3 [21, 22]. In addition, RLK independently phosphorylates the adapter molecule SLP-76 leading to PLCγ activation [23], which could explain its compensatory role in T cell activation and differentiation in the absence of ITK. Our data support a model in which selective inhibition of ITK de-represses Foxp3 expression, perhaps in part by modulating PI3K-AKT signaling, while RLK could be required to promote PLCγ-mediated T cell activation, hence playing a permissive role in T_REG_ differentiation.

While we have shown that ITK knockdown enhances the *in vitro* differentiation of human T_REG_ with functional suppressive capacity *in vitro*, we cannot discriminate whether ITK knockdown affects T_REG_ suppression on a per-cell basis. In fact, even though initial ITK knockdown promotes T_REG_ differentiation, sdRNA content is expected to decrease due to T cell proliferation and/or sdRNA degradation, hence allowing ITK re-expression at later stages (such as during our T_REG_ suppression assays). Thus, while our data show that ITK knockdown increases T_REG_ frequencies, we cannot determine whether ITK is dispensable for suppressive function after T_REG_ have been generated. The latter is a potentially important question, because a previous report showed that T_REG_ from ITK-deficient mice did not protect in a colitis model [7], although another study found that *ex vivo*-induced ITK^-/-^ T_REG_ efficiently abrogated inflammation in a similar model [5].

The reciprocal effects of ITK on effector T cell and T_REG_ differentiation makes this a potentially attractive target in autoimmune and inflammatory diseases. However, the lack of small molecule inhibitors selective for ITK, sparing other Tec kinases such as RLK, makes this target technically challenging for drug development. In fact, our current data in human T cells plus previous data in ITK/RLK double knockout mice [1] indicate that targeting both Tec kinases might lead to excessive immunosuppression, abrogating the beneficial immunomodulatory effect of increasing T_REG_ differentiation that could be achieved by targeting ITK alone. A selective ITK inhibitor would also allow us to determine whether specific blockade of ITK kinase activity will be sufficient to emulate the positive effect on human T_REG_ observed by ITK knockdown.
